# Truncated PD1 Engineered Gas‐Producing Extracellular Vesicles for Ultrasound Imaging and Subsequent Degradation of PDL1 in Tumor Cells

**DOI:** 10.1002/advs.202305891

**Published:** 2024-01-23

**Authors:** Siyan Zhang, Yuan Liang, Panpan Ji, Rui Zheng, Fan Lu, Guangdong Hou, Guodong Yang, Lijun Yuan

**Affiliations:** ^1^ Department of Ultrasound Diagnostics Tangdu Hospital Fourth Military Medical University Xinsi Road No. 569th Xi'an 710038 P. R. China; ^2^ Department of Digestive Surgery Xijing Hospital Fourth Military Medical University Shaanxi 710032 P. R. China; ^3^ State Key Laboratory of Holistic Integrative Management of Gastrointestinal Cancers and Department of Biochemistry and Molecular Biology Fourth Military Medical University Changlexi Road No. 169th Xi'an 710032 P. R. China; ^4^ Department of Urology Xijing Hospital Fourth Military Medical University Xi'an 710032 P. R. China

**Keywords:** anti‐tumor immunity, gas‐producing extracellular vesicle, programmed death 1, programmed death ligand 1, ultrasound imaging

## Abstract

PDL1 blockade therapy holds great promise in cancer immunotherapy. Ultrasound imaging of PDL1 expression in the tumor is of great importance in predicting the therapeutic efficacy. As a proof‐of‐concept study, a novel ultrasound contrast agent has been innovated here to image and block PDL1 in the tumor tissue. Briefly, extracellular vesicles (EVs) are engineered to display truncated PD1 (tPD1) on the surface to bind PDL1 with high affinity by fusion to EV‐abundant transmembrane protein PTGFRN. The engineered EVs are then encapsulated with Ca(HCO_3_)_2_ via electroporation and designated as Gp‐EV^tPD1^, which would recognize PDL1 highly expressed cells and produce gas in the endosomes and lysosomes. On the one hand, the echogenic signal intensity correlates well with the PDL1 expression and immune response inhibition in the tumor. On the other hand, during the trajectory of Gp‐EV^tPD1^ in the recipient cells, tPD1 on the EV binds PDL1 and triggers the PDL1 endocytosis and degradation in endosomes/lysosomes in a sequential manner, and thus boosts the anti‐tumor immunity of cytotoxic T cells. In summary, Gp‐EV^tPD1^ serves as a novel ultrasound contrast agent and blocker of PDL1, which might be of great advantage in imaging PDL1 expression and conquering immune checkpoint blocker resistance.

## Graphical abstract

Extracellular vesicles (EVs) are engineered with fusion protein truncated PD1‐PTGFRN (tPD1‐PTGFRN) and then loaded Ca(HCO_3_)_2_ via electroporation, designated as Gp‐EV^tPD1^. Gp‐EV^tPD1^ would recognize PDL1 highly expressed cells and produce CO_2_ gas bubbles to realize ultrasound imaging of PDL1 and trigger PDL1 degradation.

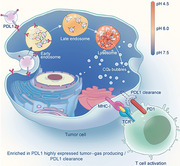

## 1 Introduction

Anti‐tumor immunotherapy has revolutionized cancer treatment. Immune checkpoint inhibitors, including inhibitors of programmed death ligand 1 (PDL1) and its receptor programmed death 1 (PD1), have been most widely used clinically, achieving over‐expected efficacy in a variety of patients with refractory, relapsed tumors.^[^ ^1^ ^]^ Although immunotherapy has had remarkable results, not all patients will benefit from it,^[^ ^2^ ^]^ attributed to the inherent temporal, and spatial heterogeneity of PD1/PDL1 expression within the tumor. Moreover, with the disease progression, the PD1/PDL1 expression profile dynamically changes. Therefore, noninvasive real‐time detection of PDL1 expression will be meaningful to predict therapeutic efficacy and adjust treatment regimens.

Molecular imaging by different modalities, such as magnetic resonance imaging, X‐ray, computed tomography (CT), optical imaging, and ultrasound, coupled with molecular probes to identify tumor‐specific markers, is expected to be able to probe the molecular events in the tumor. Specifically, ultrasound molecular imaging with antibody/ligand decorated microbubbles (MBs) has the advantages of high spatial resolution, non‐toxic, and suitability for repeated examination.^[^ ^3^ ^]^ Due to their large size, traditional MBs are restricted in the blood flow and can only be used for vasculature imaging. To improve the tumor vasculature penetration and make tumor cell molecular imaging available, types of targeted nanoscale ultrasound contrast agents (tnUCA) are designed.^[^ ^4^ ^]^ For example, liquid fluorine, which can undergo liquid gas phase transition, is used as the core of nano UCA. When the UCA escapes from the blood vessel and penetrates the tumor tissue,^[^ ^5^ ^]^ the small‐size nanoparticles will be triggered to transform into large‐size particles in situ through the acoustic droplet vaporization and thus be visualized.^[^ ^6^ ^]^ Similarly, hydrolysis of the side chain of polyester carbonate can generate CO_2_ bubbles, which can further expand and fuse to form large MBs,^[^ ^7^ ^]^ emerging as a new choice for ultrasound molecular imaging of cancer. However, these nanoscale ultrasound contrast agents cannot effectively respond to the US because of their poor echogenic sensitivity.^[^ ^3a^ ^]^ In addition, their stability also needs to be improved.

To this end, strategies to change the UCA outer membrane are also explored.^[^ ^4^ ^]^ As a natural membrane‐derived carrier, the extracellular vehicles (EVs) have the advantages of good stability and high biocompatibility.^[^ ^8^ ^]^ EVs are also easy to be engineered to increase their targeting through a variety of surface modification strategies.^[^ ^9^ ^]^ Recently, EVs have been used for immunomodulation in cancer therapy. For example, ICAM‐1‐decorated EV loaded with miR‐146a and Glut1, designed by Duarte‐Sanmiguel et al., can drive immunomodulation and hinder tumor progression in a breast cancer model.^[^ ^10^ ^]^ Similarly, by engineering exosomes through genetic display of both anti‐human CD3 and anti‐human HER2 antibodies, the resultant synthetic multivalent antibodies retargeted exosomes (SMART‐Exos), dually target T cell CD3 and breast cancer‐associated HER2 receptors and exhibit highly potent and specific anti‐tumor activity both in vitro and in vivo.^[^ ^11^ ^]^

**Scheme 1:**
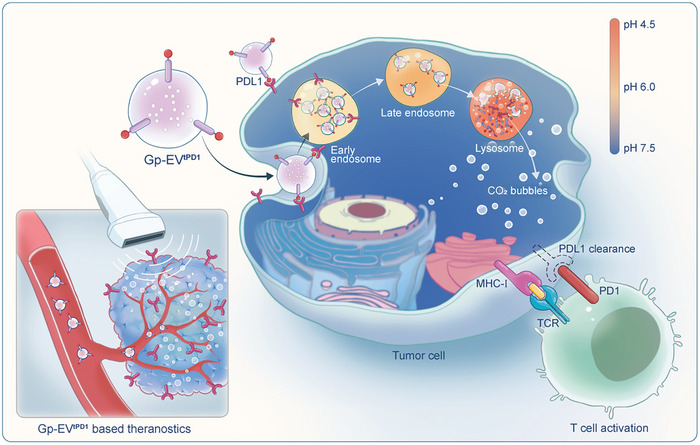
Illustration of Gp‐EV^tPD1^ based ultrasound imaging of PDL1. In Gp‐EV^tPD1^, tPD1‐PTGFRN fusion protein modification enabled the EVs to target PDL1 expressed in the tumor, and Ca(HCO_3_)_2_ loaded internally empowers the EVs with the characteristic of gas production and thus echogenic capacity when endocytosed by recipient cells. Meanwhile, Gp‐EV^tPD1^ also serves as a degrader of PDL1 and is of great advantage in conquering immune checkpoint blocker resistance.

In this study, we proposed to use EVs as vectors to construct a new ultrasound contrast agent, designated as Gp‐EV^tPD1^. The surface of EVs was modified with tPD1‐PTGFRN fusion protein, which was thus able to target PDL1 expressed in the tumor. Ca(HCO_3_)_2_ was loaded internally, giving it the characteristic of gas production when endocytosed by recipient cells. By in vitro and in vivo experiments, we have confirmed that Gp‐EV^tPD1^ serves as a novel ultrasound contrast agent to image PDL1 expression and meanwhile as a degrader of PDL1. Gp‐EV^tPD1^ should be of great advantage in imaging PDL1 expression and conquering immune checkpoint blocker resistance (**Scheme** **1**).

## 2 Results

### 2.1 Construction of PDL1‐Targeted EVs

**Figure 1:**
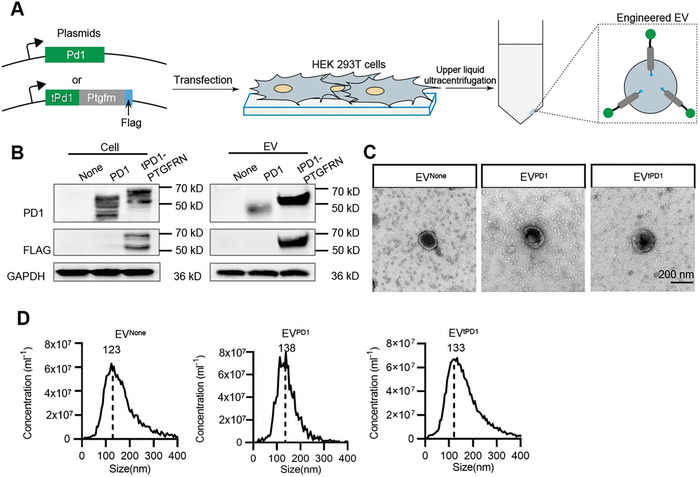
Construction and characterization of EV^tPD1^. A) Schematic illustration of the EV engineering process. B) Western blot analysis of the fusion protein in EVs derived from HEK293T cells treated as indicated. GAPDH served as a loading control. C) Representative transmission electron microscopy (TEM) image of the isolated EVs. Scale bar = 100 nm. D) The size distribution of the isolated EVs. Data shown are representative of 3 different experiments.

To construct EVs with the capacity to target/recognize PDL1, plasmids expressing full‐length Pd1 or tPd1 (truncated Pd1)‐Ptgfrn‐flag were designed and transfected into HEK293T cells, and the derived EVs were then isolated by ultracentrifugation (**Figure** **1**A). Hereafter, tPD1‐PTGFRN‐engineered EV was denoted as EV^tPD1^, PD1‐engineered EV as EV^PD1^, and EVs from control cells without treatment were denoted as EV^None^. Western blot analysis of the inclusive and exclusive exosomal markers further revealed that the isolated EVs were mainly exosomes (Figure S1, Supporting Information). In addition, overexpression of PD1 or tPD1‐PTGFRN in both the donor cells and the derived EVs was also confirmed by Western blot analysis (Figure 1B). Notably, tPD1‐PTGFRN in EV^tPD1^ was much higher than PD1 in EV^PD1^, suggesting that there were more tPD1‐PTGFRN displayed per EV, which is consistent with the previous findings that PTGFRN is one of the most abundant proteins found in the EVs.^[^ ^10^ ^]^ To explore whether tPD1‐PTGFRN‐engineering changed the size and morphology of the derived EVs, the morphology and size distribution of isolated EVs were characterized by transmission electron microscope and nanoparticle tracking analysis, respectively. Both EV^PD1^ and EV^tPD1^ had similar morphology and size as the non‐modified EV^None^ (Figure 1C,D).

**Figure 2:**
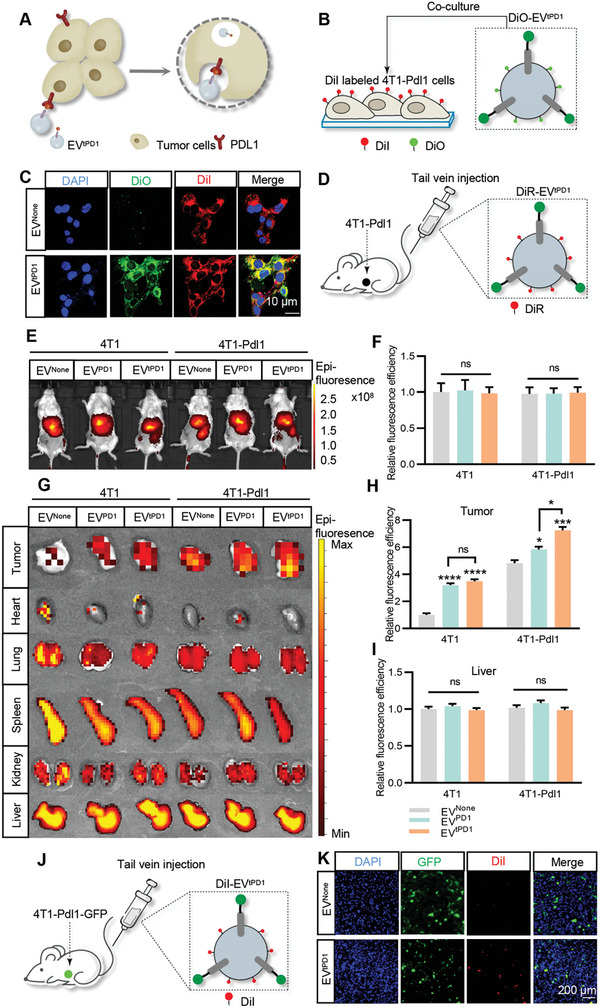
EV^tPD1^ has a high affinity to the PDL1 overexpressing cells. A) Schematic illustration of the endocytosis process of control, EV^PD1^ or EV^tPD1^. B,C) Schematic illustration and immunofluorescence stain analysis of the intracellular accumulation of EVs in 4T1‐Pdl1 cells. Control, EV^PD1^ or EV^tPD1^ were labeled by DiO (green), and 4T1‐Pdl1‐GFP cells were labeled by DiI (red). Scale bar = 50 µm. D–F) Control, EV^PD1^, or EV^tPD1^ were labeled by DiR and traced by in vivo imaging after tail vein injection. Statistical quantification of the intensity by one‐way ANOVA. G–I) Ex vivo imaging of the EV distribution in different organs. J,K) Schematic illustration and fluorescence stain analysis of the intracellular accumulation of control, EV^PD1^ or EV^tPD1^ in 4T1‐Pdl1‐GFP tumor‐bearing mouse model. EVs were labeled by DiI (red), 4T1‐Pdl1 cells were labeled by GFP (green), and nuclei were stained with DAPI (blue). Scale bar = 200 µm. Data shown are representative of 3 different experiments. ns: not significant; *P < 0.05; ***P < 0.001; ****P < 0.0001 by one‐way ANOVA.

To confirm whether tPD1 (displayed on the EV) could interact with PDL1, PDL1 containing cell lysate was incubated with EV^tPD1^, followed by immunoprecipitation. As expected, PDL1 was abundantly observed in the precipitate of EV^tPD1^(Figure S2, Supporting Information). We next verified the PDL1 targeting ability of EV^tPD1^. 4T1 cell line overexpressing PDL1 (4T1‐Pdl1) was constructed by lentivirus infection, and qPCR and Western blot analysis confirmed the high expression efficiency of PDL1 (Figure S3, Supporting Information). Fluorescence‐labeled EVs were then incubated with control or 4T1‐Pdl1 cells. As expected, more EV^tPD1^ was found uptaken by 4T1‐Pdl1 cells, in comparison with control EVs (**Figures** **2**C and S4, Supporting Information). Similarly, 4T1‐Pdl1 cells were also found taking up more EV^tPD1^ than 4T1‐Ctrl cells (Figure S4, Supporting Information). To further observe the in vivo distribution, DiI or DiR labeled EV^tPD1^ was injected in mice and tracked. As expected, EV^tPD1^ was highly accumulated in 4T1‐Pdl1 tumors (Figure 2D–I; Figures S5 and S6, Supporting Information). Immunofluorescence of tumor sections further revealed abundant localization of EV^tPD1^ in 4T1‐Pdl1‐GFP cells (Figure 2J,K; Figure S7, Supporting Information). Moreover, no organ toxicity was observed via H&E staining (Figure S8, Supporting Information).

Gp‐EVs could enter cells through multiple ways. To further determine the proportion of endocytosis‐mediated uptake of EVs, 4T1‐Pdl1 cells were additionally treated with clathrin‐mediated endocytosis (CME) inhibitor Pistop‐2 or anti‐PDL1 antibody or the combination. Pistop‐2 or anti‐PDL1 antibody or the combination treatment significantly decreased the uptake of EV^tPD1^. Simultaneous treatment with anti‐PDL1 and Pistop‐2 reduced the uptake of EV^tPD1^ to 4.19%, indicating that EV^tPD1^ enters the cells mainly via receptor‐ligand interaction mediated endocytosis (Figure S9, Supporting Information).

### 2.2 Echogenicity of the Gas Producing‐EV Gp‐EV^tPD1^

**Figure 3:**
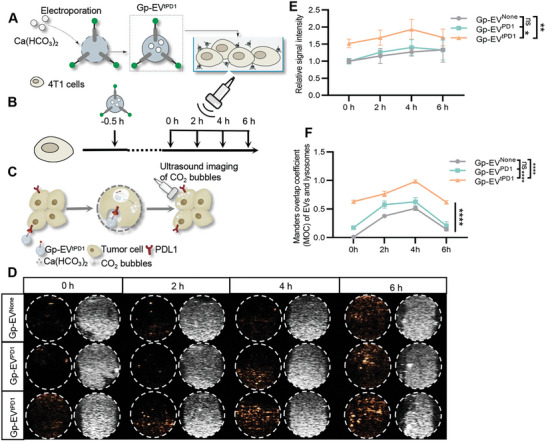
Time‐dependent in vitro US imaging. A) Schematic illustration of in vitro US image procedure. B) Schematic illustration of the experimental procedure. C) Schematic illustration of the gas generation process. D,E) Handmade agar gel phantom was used for in vitro US imaging. Time‐dependent in vitro US images of Gp‐EVs over time in saline at 37 °C. The US images were normalized by subtracting the water intensity from the phantom signal. F) Quantification of immunofluorescence co‐localization between lysosomes and Gp‐EVs. Statistical quantification of the intensity by two‐way ANOVA. ns: not significant; *P < 0.05; **P < 0.01; ****P < 0.0001. Data shown are representative of 3 different experiments; 3 mice in both groups.

To construct gas producing‐EVs (Gp‐EVs), Ca(HCO_3_)_2_ was loaded into EVs by electroporation, with tPD1‐PTGFRN engineered Gp‐EVs denoted as Gp‐EV^tPD1^, PD1‐engineered Gp‐EV as Gp‐EV^PD1^, and Gp‐EV with no gene engineering denoted as Gp‐EV^None^ and served as control. Theoretically, Ca(HCO_3_)_2_ in Gp‐EVs will react with H^+^ in lysosomes and generate CO_2_ bubbles when endocytosed by the recipient cells. To monitor the echo properties of Gp‐EV^tPD1^, 1–3% (w/v) of handmade agar gel phantom was used to contain co‐cultured Gp‐EVs and 4T1 cells, while ultrasound (US) image was performed by Vevo 2100 with 25 MHz probe (**Figure** **3**A–C). Cell viability assay was performed to observe the cytotoxicity of different Gp‐EVs. Notably, control Gp‐EV or Gp‐EV^tPD1^ treatment did not change the cell viability (Figure S10, Supporting Information), suggesting the high safety of the Gp‐EVs.

After endocytosis by the recipient cells, Gp‐EV presented a gradual increase in echo signals from 0 h to 4 h, with a slight decline at 6 h (Figure 3D,E). Consistently, Gp‐EV colocalized with the lysosomes in a time‐dependent manner, reaching the highest at 4 h and declining since then (Figure S11, Supporting Information; Figure 3F). Compared with the Gp‐EV^None^ and Gp‐EV^PD1^ groups, the echogenic signal and fluorescence intensity in the Gp‐EV^tPD1^ group were much higher (Figure 3D–F), which was consistent with the finding that more Gp‐EV^tPD1^ was taken up by the 4T1‐Pdl1 cells. Similar time‐dependent echogenic signal changes were observed in the syngeneic 4T1 mouse model (Figure S12, Supporting Information). The results indicated that the echogenic signal produced from Gp‐EV^tPD1^ in the recipient cells could be monitored by ultrasound imaging.

**Figure 4:**
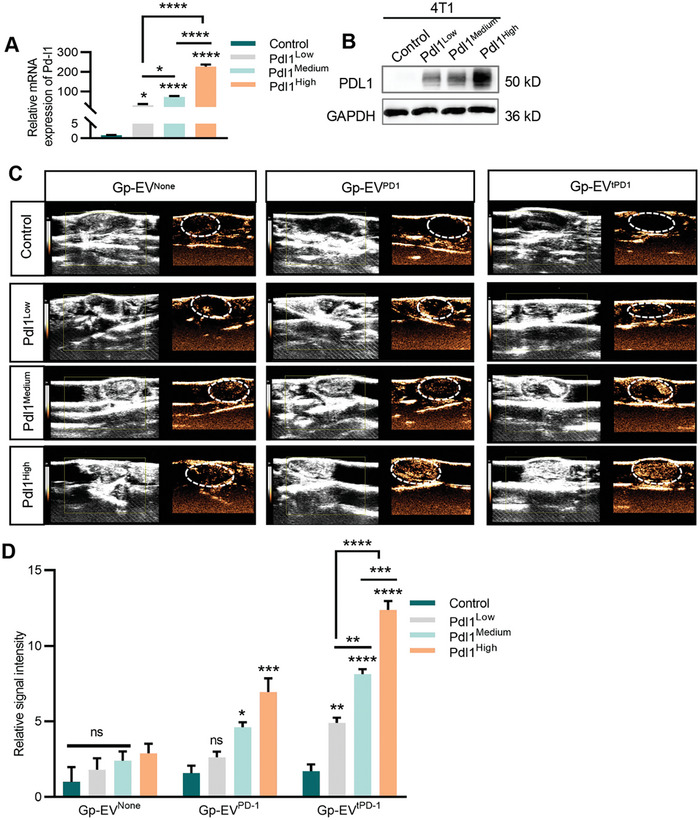
Ultrasound imaging of PDL1 expression via Gp‐EV^tPD1^. A) qPCR analysis of Pdl1 mRNA expression after infection. 4T1 cells were infected by different titers of lentivirus co‐expressing GFP and Pdl1 to generate cell lines with different PDL1 expression levels, named 4T1‐Pdl1^Low^, 4T1‐Pdl1^Medium^ and 4T1‐Pdl1^High^ respectively. PDL1 expression level was identified by GFP fluorescence intensity. B) Western blot analysis of PDL1 expression. GAPDH served as a loading control. C,D) In vivo US image of control, PD1 engineered Gp‐EV or Gp‐EV^tPD1^ in PDL1 expression gradient model. Statistical quantification of the intensity by one‐way ANOVA. Data shown are representative of 3 different experiments; 3 mice in each group. ns: not significant; *P < 0.05;** P < 0.01; ***P < 0.001; ****P < 0.0001 by one‐way ANOVA.

To further explore whether Gp‐EV^tPD1^ could monitor PDL1 expression level in vivo, 4T1 cells with gradient PDL1 expression levels, denoted as 4T1‐Pdl1^Low^, 4T1‐Pdl1^Medium^, 4T1‐Pdl1^High^ were established. GFP fluorescence, qPCR, and Western blot analysis confirmed the PDL1 expression gradients (Figure S13, Supporting Information; **Figure** **4**A,B). Gp‐EVs were injected into the syngeneic mouse models inoculated with 4T1, 4T1‐Pdl1^Low^, 4T1‐Pdl1^Medium^, and 4T1‐Pdl1^High^ via tail veins respectively, and 4 h later ultrasound imaging was performed. The results showed that the ultrasound echogenicity produced by Gp‐EV^tPD1^ increased with the increment of PDL1 expression, indicating that Gp‐EV^tPD1^ can monitor the expression of PDL1. Similarly, the ultrasonic echogenic intensity from Gp‐EV^PD^ also increased with the PDL1 enhanced, though with a much lower magnitude. In contrast, the ultrasonic echogenic intensity from Gp‐EV^None^ had no significant changes among different groups with gradient PDL1 expression (Figure 4C,D). Together, these data showed that Gp‐EV^tPD1^ could serve as a UCA for ultrasound imaging of PDL1 in the tumor.

### 2.3 Therapeutic Efficacy of Gp‐EV^tPD1^

**Figure 5:**
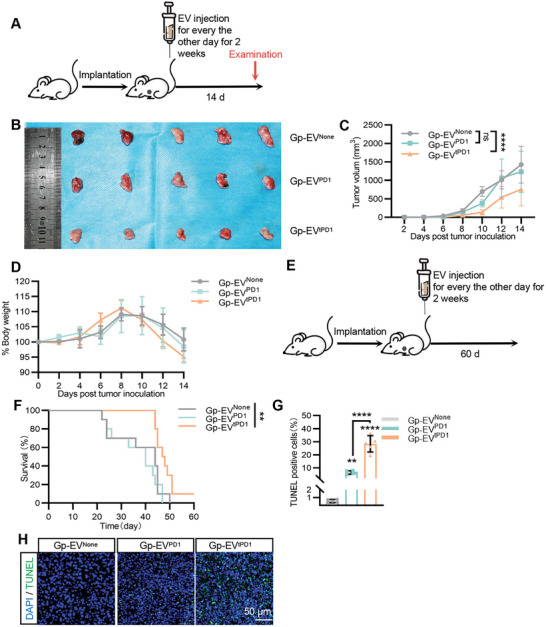
Anti‐tumor effects of Gp‐EV^tPD1^ in vivo. A) Schematic illustration of the experimental procedure. 4T1‐Pdl1^High^ cells were injected orthotopically into the right mice gland fat pad of 6–8‐week‐old balb/c mice. Control, PD1‐engineered Gp‐EV, or Gp‐EV^tPD1^ were injected via tail veins as the treatment every 2 days. 14 days after inoculation of the tumor cells, tumor tissue was harvested for the following experiments. B) Images of tumor tissues from different EV treatments. C) Tumor growth curves of orthotopically implanted 4T1‐Pdl1^High^ tumors following treatment with control, PD1 engineered Gp‐EV or Gp‐EV^tPD1^. D) Body weight curve of 4T1‐Pdl1^High^ tumor‐bearing mice with treatment as indicated. E,F) Survival curves of 4T1‐Pdl1^High^ tumor‐bearing mice treated with treatment as indicated. G,H) Fluorescent TUNEL (indicates apoptotic cells) stained of 4T1‐Pdl1^High^ tumor tissue in mice treated as indicated at the end of the experiments. Scale bar = 50 µm. Data shown are representative of 5–7 mice in each group. ns: not significant; **P < 0.01; ****P < 0.0001 by one‐way ANOVA or two‐way ANOVA.

To explore whether Gp‐EV^tPD1^ could also inhibit tumor growth, Balb/c mice inoculated with 4T1‐Pdl1^High^ cells were treated with Gp‐EV^None^, Gp‐EV^PD1^, or Gp‐EV^tPD1^ every other day for 2 weeks, beginning at 4 days after tumor inoculation. As expected, Gp‐EV^tPD1^ treated mice displayed smaller tumor volumes (**Figure** **5**A,B; Figure S14A, Supporting Information). The body weight of 4T1‐Pdl1^High^ tumor‐bearing mice had no significant differences among groups, while Gp‐EV^tPD1^ treatment prolonged animal survival time (Figure 5C–F, Figure S14B, Supporting Information). TUNEL staining showed that Gp‐EV^tPD1^ treatment elicited extensive cell death in tumors, suggesting the robust anti‐tumor effect of Gp‐EV^tPD1^. (Figure 5G,H). To further confirm the therapeutic effects dependent on T cells, we then investigated whether Gp‐EV^tPD1^ could achieve similar therapeutic effects in T cell absent Balb/c nude mice. No significant change in tumor volumes was observed (Figure S15, Supporting Information), indicating therapeutic effects of Gp‐EV^tPD1^ are T cell activity‐dependent.

### 2.4 Gp‐EV^tPD1^ Triggers PDL1 Degradation and Elicits Anti‐Tumor Immunity

**Figure 6:**
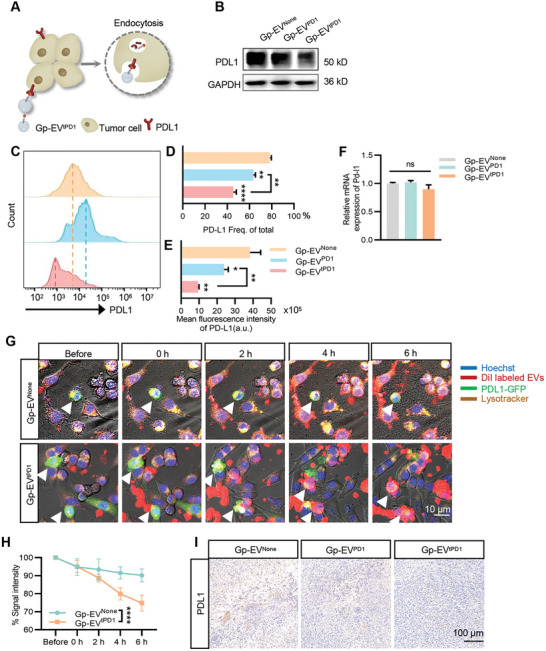
Gp‐EV^tPD1^ triggers PDL1 degradation in tumor cells. A) Schematic illustration of the degradation process of PDL1 via receptor‐mediated endocytosis caused by the binding of PDL1 and tPD1‐PTGFRN (which contained extracellular domain of PD1) after tPD1‐PTGFRN engineered Gp‐EVs treatment. B) Western blot analysis of PDL1 expression. GAPDH served as a loading control. C–E) Flow cytometry analysis of PDL1 expression in tumor tissues. The PDL1 expression levels were evaluated by fluorescence frequency of total and mean fluorescence intensity (MFI). Statistical quantification of the intensity by one‐way ANOVA. F) qPCR analysis of Pdl1 mRNA expression in tumor tissues. G,H) The live cell station was used to monitor the PDL1 degeneration dynamically. PDL1‐GFP (green) was overexpressed in 4T1 cells; Gp‐EV^None^ or Gp‐EV^tPD1^ were labeled with DiI (red); Lysosomes were labeled by LysoTracker Deep Red (yellow), and nuclei were stained with Hoechst (blue). Scale bar = 10 µm. Statistical quantification of the intensity by one‐way ANOVA. I) Immunohistochemical images showing the expression of PDL1 (brown) in tumor tissue. Scale bar = 100 µm. Data shown are representative of 5–7 mice in each group. ns: not significant; **P < 0.01; ***P < 0.001; ****P < 0.0001 by one‐way ANOVA or two‐way ANOVA.

To explore the potential degradation‐promoting effects of Gp‐EV^tPD1^ on PDL1 expression, 4T1‐Pdl1^High^ cells were incubated with Gp‐EV^None^, Gp‐EV^PD1^, or Gp‐EV^tPD1^ and expression of PDL1 was then analyzed by western blot. Gp‐EV^tPD1^ significantly decreased PDL1 expression (**Figure** **6**A,B). Flow cytometry showed similar results (Figure 6C–E). In contrast, Gp‐EV^tPD1^ did not change the Pdl1 mRNA expression, as determined by qPCR analysis (Figure 6F). To further confirm whether Gp‐EV^tPD1^ triggered PDL1 degradation in the lysosomes, PDL1‐GFP fusion protein was overexpressed in 4T1 cells by transfection. After the addition of Gp‐EV^tPD1^, PDL1‐GFP expressed in 4T1 cells was gradually induced to degradation in the lysosomes (Figure 6G,H, Supplementary Video). Consistent with the in vitro data, immunohistological analysis also showed that Gp‐EV^tPD1^ treatment could decrease the PDL1 expression in the tumor (Figure 6I). To explore whether Gp‐EV^tPD1^ treatment could also decrease the expression of PDL1 in immune cells, RAW 264.7 cells were treated with Gp‐EV^tPD1^. Consistent with the effects on tumor cells, the western blot showed that Gp‐EV^tPD1^ decreased PDL1 expression in a similar manner (Figure S16, Supporting Information).

**Figure 7:**
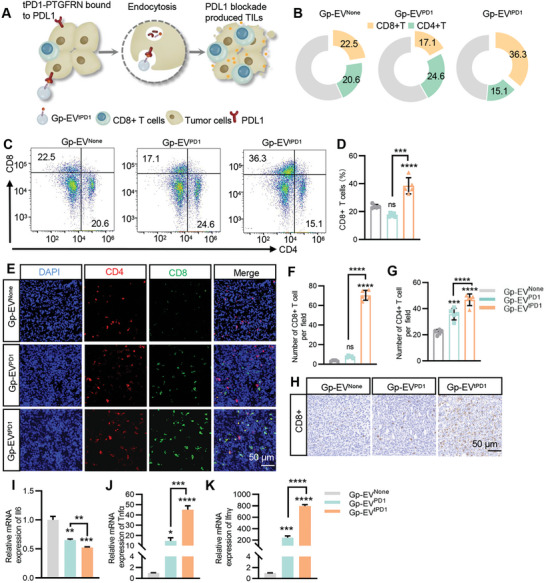
Stimulation of anti‐tumor immunity by Gp‐EV^tPD1^. A) Schematic illustration of Gp‐EV^tPD1^ mediated PD1/PDL1 blockade unleashes CD8+ T cell to attack tumor cells. B–D) Flow cytometry analysis of the T cell distribution in the 4T1‐Pdl1^High^ tumor tissues with treatment as indicated. Cell suspensions prepared from tumors were examined by flow cytometry to assess T cell infiltration. E–G) Immunofluorescence stain analysis of the amount of CD8+ T (green) and CD4+ T (red) cells in the tumors with treatment as indicated. Statistical quantification of the intensity by one‐way ANOVA. Scale bar = 50 µm. H) Immunohistochemical images showing the expression of CD+ T cells (brown) in tumor tissue. Scale bar = 50 µm. qPCR analysis of I) Il‐6, J) Ifn‐γ, K) Tnf‐α mRNA expression in tumor tissues. Statistical quantification of the intensity by one‐way ANOVA. Statistical quantification of the intensity by one‐way ANOVA. Scale bar = 50 or 5 µm. Data shown are representative of 5–7 mice in each group. ns: not significant; **P < 0.01; ***P < 0.001; ****P < 0.0001 by one‐way ANOVA.

Theoretically, CD8+ T cells would be activated if PDL1 is blocked or inhibited.^[^ ^13^, ^14^ ^]^ Consistently, with the degradation of PDL1 induced by Gp‐EV^tPD1^, CD8+ T cell infiltration was significantly increased, as determined by flow cytometry and immunostaining (**Figure** **7**A–H, Figure S17, Supporting Information). In addition, the pro‐inflammatory cytokines IFNγ, and TNFα, were significantly increased after Gp‐EV^tPD1^ treatment. In contrast, there was a marked decline of IL6 in the Gp‐EV^tPD1^ treatment group (Figure 7I–K, Figure S18, Supporting Information). The decline of IL6 might also contribute to the increase of IFNγ, as it is reported that IL6 could inhibit IFNγ expression in breast cancer.^[^ ^15^ ^]^ All of the data suggested that Gp‐EV^tPD1^ treatment would elicit anti‐tumor immunity by blocking PD1/PDL1 axis.

## 3 Discussion

In this study, Gp‐EV^tPD1^ with tPD1 was displayed on the surface, and Ca(HCO_3_)_2_ encapsulated inside was designed and engineered as a novel ultrasound contrast agent to image PDL1 expression and meanwhile as a degron of PDL1. The surface displayed tPD1 was able to recognize PDL1 expressed in the tumor and thus determine the uptake efficiency and amount of gas production. Monitoring of the gas production could predict the PDL1 expression in the tumor. Meanwhile, during the endocytosis of the EVs, the tPD1 would elicit the degradation of PDL1 and thus boost anti‐tumor immunity. Together, Gp‐EV^tPD1^ has great advantages in imaging PDL1 expression and conquering immune checkpoint blocker resistance.

In terms of real‐time monitoring, molecular imaging has more advantages than canonical pathological examination. MBs, with a stabilizing shell (e.g., phospholipids, proteins, synthetic polymer or surfactants) and low‐diffusive perfluorocarbon (PFC) gas core, with a diameter of 1–10 µm, are usually applied for ultrasonic enhanced imaging.^[^ ^4^ ^,^ ^16^ ^]^ When armed with antibodies or specific ligands, the targeting UCA could recognize specific molecular targets expressed on the endothelial cell surface and thus be utilized for molecular imaging. Notably, given the large scale of MBs, only the molecular targets in the blood vessel wall could be imaged, which limits their clinical application. Even for the tumors, large‐scale UCA is still not allowed to penetrate through the endothelium. Furthermore, the low stability of commercial MBs makes them difficult to remain echogenic after penetration.^[^ ^4^ ^]^ The nanoscale NPs can pass through the tumor vasculature and directly target the tumor cells. To this end, tumor tumor‐targeting nanoscale ultrasound contrast agent called tnUCA was designed and played an indispensable role in ultrasound theranostic.^[^ ^17^ ^]^ It should be noted that the nanoscale NPs cannot effectively respond to the US because of their poor echogenic sensitivity, resulting in a significant decrease in image contrast compared with MBs.^[^ ^3a^ ^]^ Mechanistically, the sub‐micrometer PFC droplets exhibited weak US‐scattering performance because of their low impedance and compressibility, behaving as linear Rayleigh scatterers.^[^ ^6a^ ^,^ ^18^ ^]^ In addition, these synthetic tnUCAs are not stable enough after penetration and biosafety is also another concern.

The Gp‐EV^tPD1^ we proposed here represents a novel tnUCA with a brand new concept. It is well established that EVs could break types of barriers via the process called transcytosis.^[^ ^19^ ^]^ The nanoscale of Gp‐EV^tPD1^ and the intrinsic characteristics of EV allow it to penetrate into the tumor with high efficiency, making tumor cell ultrasound molecular imaging possible. In the recipient cells, Ca(HCO_3_)_2_ loaded in the core produces CO_2_ bubbles in the acidic environment of lysosomes in a gradual manner, which in turn will be fused to large CO_2_ bubbles. Notably, cancer cells actively uptake hundreds of EVs^[^ ^20^ ^]^ and thus would produce gas continuously in a relatively long period. In other words, the gas is actively produced in the recipient cells and strong enough to be visualizable and monitorable by ultrasound, which is totally different from the pre‐existing bubbles or ADV‐mediated bubbles within the contrast agents. tPD1‐PDL1 interaction facilitates the uptake of EV by recipient cells, and cells with high expression of PDL1 efficiently uptake more Gp‐EV^tPD1^. With the Gp‐EV^tPD1^, ultrasound imaging could thus detect PDL1 expression in the tumor tissue, though the detailed parameters and standards corresponding to PDL1 expression level still need to be explored. In the context of immunotherapy, examination of CTLA4, PD1, and other checkpoint molecules is also important and developed multiple strategies.^[^ ^21^ ^]^ Recently, the serial collapse of targeting microbubbles with distinct acoustic pressures makes ultrasound molecular imaging for multiple biomarkers practical.^[^ ^22^ ^]^ In terms of the Gp‐EV, imaging of the different molecules with intervals, such as every other day, could also be feasible as the injected EVs could be totally cleared within 48 h.^[^ ^23^ ^]^ Up to now, biosafety has been the most common concern that hinders the clinical translation of targeted contrast agents, especially for repeated use. For example, the positive surface charge of lipid nanoparticles (LNPs) makes them tend to accumulate and aggregate within the negatively charged extracellular matrix,^[^ ^24^ ^]^ and exogenous fabricated nanoparticles could induce oxidative stress.^[^ ^25^ ^]^ The excellent biosafety of EVs makes Gp‐EV^tPD1^ clinical translation promising, while the low yield might be the bottleneck that needs to be broken.

EV‐based immunotherapies have been extensively explored recently, especially in breast cancer. Duarte‐Sanmiguel et al. designed ICAM‐1‐decorated EV loaded with miR‐146a and Glut1, which could drive immunomodulation and hinder tumor progression in a breast cancer model.^[^ ^10^ ^]^ Shi et al. developed a synthetic multivalent antibodies retargeted exosome (SMART‐Exo) displaying both anti‐human CD3 and anti‐human HER2 antibodies, and the SMART‐Exos dually targeting T cell CD3 and breast cancer‐associated HER2 receptors for immune‐activation.^[^ ^11^ ^]^ EVs were also used to pack GSDMD‐N mRNA to induce cell pyroptosis in HER + breast cancer,^[^ ^26^ ^]^ in turn the immunogenic pyroptosis induced a robust immunotherapy. Moreover, immune cell‐derived EVs and tumor cell‐derived EVs were used as antigen sources or adjuvant carriers to boost anti‐cancer immune response.^[^ ^27^ ^]^ We here have proposed a novel EV‐based immunomodulatory strategy. The interaction between PD1 on T cells and PDL1 on tumor cells or other stromal cells in trans triggers inhibitory signaling to attenuate T cell responses, and these inhibitory signals are blocked usually by the usage of antibodies against PD1 or PDL1.^[^ ^28^ ^]^ Once the interaction between PD‐L1 and PD1 is blocked, pre‐existing anti‐tumor T cells can quickly restore their immune function. Immunotherapies that target the PDL1/PD1 axis have demonstrated unprecedented efficiency in the treatment of a variety of human cancers.^[^ ^29^ ^]^ PDL1 is expressed in 20% to 50% of human cancers.^[^ ^30^ ^]^ In addition to T cell inhibition, PDL1 could also suppress IFNβ mediated cytotoxicity and help tumor cells develop resistance to type I and II interferon.^[^ ^31^ ^]^ The anti‐PDL1 antibody can increase lysosomal PDL1 degradation and ultimately decrease the PDL1 level in tumor cells.^[^ ^32^ ^]^ In the engineered Gp‐EV^tPD1^ we proposed here, the extracellular segment of PD1 (tPD1) was fused to PTGFRN and thus enriched on the surface of EVs as the PTGFRN was identified as the most abundant membrane protein of EVs.^[^ ^12^ ^]^ Gp‐EV^tPD1^ accumulated in the tumor not only reflects PDL1 abundance, but also increases lysosomal degradation of PDL1, playing a role similar to anti‐PDL1 antibody. As to the mechanism of how tPD1 on the EV triggers the degradation of PDL1, we prefer the model that endocytosis of Gp‐EV^tPD1^ will simultaneously deliver the PDL1 bound by the EV to the lysosomes. As we know, the cytokine IFNγ released by CD8+ T cells plays an important role in the tumor immune cycle, and could also increase the expression of PDL1,^[^ ^33^ ^]^ suggesting that persistent blockade of PDL1 might be needed in the clinical setting. Different from PDL1 antibodies, the degradation of PDL1 by the proposed strategy has superior advantages and would be an alternative for ICB blocker therapy, especially in the context of resistance and repeat use.

It is important to note that there are some limitations of this study. For example, PDL1 is only one of markers responsible for the response to immune checkpoint blockade. Imaging more markers simultaneously with ultrasound is necessary for precision prediction and could be the future direction. In addition, the dose of EVs and the time point for diagnosis need to be optimized, and the correlation between echo signals and PDL1 expression needs to be clarified. For the therapeutic purpose, it is better to compare the efficacy of the proposed strategy to PDL1 or PD1 antibody, which is a prerequisite for the potential clinical application.

## 4 Conclusion

In summary, the engineered EVs designated as Gp‐EV^tPD1^ could recognize PDL1 highly expressed cells and produce gas in the endosomes and lysosomes of the recipient cells. During the trajectory of Gp‐EV^tPD1^ in the recipient cells, tPD1 on the EV binds PDL1 and triggers the PDL1 endocytosis and degradation in endosomes/lysosomes in a sequential manner, and thus boosts the anti‐tumor immunity of cytotoxic T cells. Meanwhile, the produced gas signal in the recipient cells could be visualized by ultrasound, reflecting the expression of PDL1. Together, Gp‐EV^tPD1^ serves as both a novel ultrasound contrast agent and degron of PDL1, which might be of great advantage in imaging PDL1 expression and conquering immune checkpoint blocker resistance.

## 5 Experimental Section

### Cell Culture

HEK293T, RAW264.7, 4T1 murine breast carcinoma cells were cultured in Dulbecco's modified eagle medium (DMEM) or Roswell Park Memorial Institute (RPMI)−1640 medium with 10% fetal bovine serum and 1% penicillin‐streptomycin (HyClone, GE, Logan, Utah, USA). Cells were grown at 37 °C in a 5% CO_2_ and were changed with fresh medium every other day. For in vitro treatment of EVs, about 10 µg EVs (at protein concentration) per well in a 6‐well plate were used. To block CME or PDL1 mediated endocytosis, 4T1‐Pdl1 cells were additionally treated with anti‐PDL1 antibody (7.5 µg mL^−1^), Psitop‐2 (25 µm), or combination before EV treatment.

### Cell Transfection

Full‐length Pd1 or tPd1‐Ptgfrn (extracellular region of PD1 fused with Ptgfrn fragment) expressing plasmids were designed (detailed sequences in Table S1, Supporting Information) and constructed in Genscript. HEK293T cells were seeded in 6‐well plates 24 h before the experiment, according to the manufacturer's protocol. EV (mainly exosomes) packaging cells (HEK293T) were transfected with Pd1 or tPd1‐Ptgfrn plasmids by Lipofectamine 2000. Briefly, 4 µg plasmid and 8 µL lipofectamine 2000 (Invitrogen, Carlsbad, CA, USA) were diluted with serum‐free DMEM culture medium, and then mixed before addition to the cells. Cells were additionally cultured in a routine medium at 37 °C, 5% CO_2_ for gene expression analysis, or in a serum‐free medium for EVs isolation.

### Lentivirus Infection

Control lentivirus and the lentivirus expressing PDL1 or PDL1‐GFP were generated by GENE Shanghai Company. 24 h before the experiment, the cells were seeded into a 6‐well plate. When the cell confluence was between 20% and 50%, the infection was performed. The virus was added to the cells together with polybrene (5% per well µg mL^−1^). The medium was changed into culture medium ≈6–18 h later. For production of 4T1 cells with different levels of PDL1, different titers of virus were used and PDL1 expression was monitored by GFP expression.

### Animal Housing

Adult female Balb/c mice or Balb/c nude mice (6−8 weeks old, 20−22 g) were purchased from the Animal Center of the Fourth Military Medical University (Xi'an, China) and housed at a constant room temperature under a 12 h/12 h light/dark cycle. Mice were housed less than five per cage and allowed free access to food and water. All animal studies were approved by the Institutional Animal Care and Use Committee.

### Tumor Models and Treatments

To generate mouse tumor models, 4T1 cells (control or with Pdl1 overexpressed as indicated) were trypsinized and injected orthotopically (2 × 10^6^ cells per mouse) into a single mammary fat pad of Balb/c mice. Gp‐EV^None^ as the control, Gp‐EV^PD1^, and Gp‐EV^tPD1^ (5 µg EV g^−1^ body weight) were injected every other day since day 2 after tumor inoculation. The treatments were administered via a lateral tail vein every day in a volume of 150 µL. During the treatment, the tumor size was measured using Vernier calipers, and the tumor volume (*V*) was calculated using the formula:

(1) $$V=\left(1/2\right)WL^{2}$$

where *W* is width, for short axes and *L* is length, for long axes, respectively. The weights of the mice were also measured every 2 days. At the end of treatment, the mice were euthanized, and the tumor xenografts were excised, weighed, processed, and prepared for the following experiments.

### Extracellular Vesicle Preparation and EV Immunoprecipitation Assay

After 24 h culture in FBS FBS‐free medium, ultracentrifugation was applied for EV isolation. Supernatants were centrifuged at 5000 g for 10 min to remove cells and cellular debris. The supernatants were filtered through 0.22 µm filters and then ultracentrifuged at 100, 000 g for 4 h. The samples were resuspended in PBS and stored at −80 °C.

To test whether tPD1 localized outside in the engineered EVs could interact with PDL1, exosome pull down assay was performed by incubating the EV^tPD1^ with PDL1‐containing cell lysate. Then the EVs were additionally isolated and whether PDL1 was co‐pelleted was confirmed by Western blot assay.

### Extracellular Vesicle Characterization

Electron microscopy (JEM‐2000EX TEM, JEOL Ltd., Tokyo, Japan) was used to analyze the morphology of isolated EVs. Briefly, EVs were resuspended and 8 µL EV solution was dropped onto copper grids. After drying for 20 min, EVs were stained with 2% uranyl acetate for 6 min and left dry for examination. The dried grids were visualized by TEM at 80 kV. For size distribution analysis of the EVs, Nanosight was included. Samples from different sources were uniformly diluted to 500 ng mL^−1^ (at vesicle protein concentration).

### Ca(HCO_3_)_2_ Loading

Ca(HCO_3_)_2_ was loaded by electroporation. EVs were resuspended in 1 mm Ca(HCO_3_)_2_ solution in a volume ratio of 1:1000. Electroporation was performed with the condition of 110 V/940 uF for 10 cycles to guarantee the final concentration of Ca(HCO_3_)_2_ in EVs were similar as 1 mm. EVs were then incubated on ice for 30 min after electroporation.

### In Vivo Distribution Analysis

Tumor model mice were injected with fluorescent dye DiR (Invitrogen, 8 µm, 5 µg g^−1^)‐labeled EVs via a lateral tail vein for in vivo tracking. EVs were incubated with the dye at 37 °C for 5 min followed by 4 °C for 15 min, washed with PBS, and re‐collected by ultracentrifugation, then resuspended in PBS. For ex vivo imaging, mice were injected with DiR‐labeled EVs via tail veins and sacrificed before imaging. 12 h after injection, the mice in or ex vivo imaging was performed (IVIS Lumina II in vivo imaging system).

For analysis of EV distribution in tissue, mice were injected with DiI (Invitrogen, 8 µm, 5 µg g^−1^)‐labeled EVs and harvested for tissue sectioning. Sections were fixed with 4% paraformaldehyde for 15 min and then stained with DAPI (Sigma). The fluorescence signals for the labeled EVs and the blue nuclei were visualized by confocal laser microscopy (A1R; Nikon, Tokyo, Japan).

### Western Blot

The samples were homogenized using RIPA buffer (Sigma Aldrich) mixed with a protease inhibitor cocktail and a phosphatase inhibitor (Complete Mini, Roche, Germany). A Pierce BCA Protein Assay Kit (Thermo Fisher Scientific, Waltham, USA) was used to determine the protein concentration. Protein samples (10 µg per lane) were separated by 12% (v/v) discontinuous sodium dodecyl sulfate‐polyacrylamide gel electrophoresis (SDS‐PAGE). The membranes were blocked in 5% of skim milk diluted in 0.1% Tween 20/Tris‐buffered saline (TBS‐T) for 1 h at room temperature, followed by incubation with primary antibodies (Anti‐DDDDK tag antibody, Abcam, USA; PD1 antibody, Abcam, USA; PDL1 antibody, Abcam, USA; GAPDH polyclonal antibody, Proteintech Inc., USA; GM130 antibody, Abcam, USA; TSG101 antibody, Abcam, USA; CD81 antibody, Abcam, USA) at 4 °C overnight. On the 2nd day, the membrane was washed in TBS‐T for 5 min × 3, following incubated with corresponding secondary antibodies for 2 h at room temperature. Visualization was performed using an enhanced chemiluminescence method under standard protocols (ECL Plus; Thermo Fisher Scientific, USA). ImageJ (National Institutes of Health, Bethesda, Maryland, USA) was used for optical density assessment.

### Quantitative PCR

Total RNA from EVs, cells, or tissues was extracted by TRIzol reagent (Invitrogen, Carlsbad, CA, USA). The RNA was reverse transcribed to cDNA using the PrimeScript first‐strand cDNA synthesis kit (Takara, Dalian, China). The reverse transcription process used a 20 µL system as instructed. The relative gene expression of mRNA was analyzed using Roche FS Universal SYBR Green Master (Roche, Switzerland). The real‐time PCR was performed using SYBR green system. The relative expression of target mRNA was calculated with the 2‐ddCt method. The detailed primer sequences are listed in Table S2, Supporting Information.

### Cytokine ELISA

The concentration of the following cytokines in the brain lysates was determined using ELISA kits according to the manufacturer's instructions: IL6 (Thermo Fisher Scientific, cat# KMC0061), IFNγ (Thermo Fisher Scientific, cat# KMC4021), TNFα (Thermo Fisher Scientific, cat# BMS607‐3).

### Fluorescence Staining

The sections were incubated overnight at 4 °C with immunofluorescent antibodies against CD4 (Abcam, USA, 1:100) and CD8 (Abcam, USA, 1:100). After washing with PBS for 5 min × 3; the sections were incubated with Cy3 or FITC labeled secondary antibody (1:200; Sigma) for 2 h at room temperature. LysoTracker Red or Deep Red (Invitrogen, USA, 75 nm) were used to trace lysosomes. Cells or sections were incubated at 37 °C for 2 h. The fluorescence signal was confirmed by confocal laser microscopy (A1R; Nikon, Tokyo, Japan).

### Flow Cytometry

Tumors were surgically removed and immediately placed in a sterile 4 °C precooled DMEM medium. The tumor tissue was cut as much as possible, and transferred to DMEM medium containing 0.5 mg mL^−1^ collagenase IV. The mixture was then kept shaking at 200 rpm, 37 °C for 90 min. After filtering through a 200 mesh sieve, the medium was centrifuged at 800 g, 4 °C for 5 min to obtain the pellet. To remove red blood cells, the pellet was resuspended by ACK Lysis Buffer (Beyotime, China) and incubated at room temperature for 5 min. The isolated cells were then resuspended in a staining buffer and stained as instructed. Anti‐mouse CD3‐PE (Biolegend, USA), anti‐mouse CD45‐FITC (Biolegend, USA), anti‐mouse CD4‐APC (Biolegend, USA), and anti‐mouse CD8‐PerCP Cy5.5 (Biolegend, USA) were used. Flow cytometry was performed by Beckman CytoFLEX (Beckman, USA) and analyzed using CytExpert and FlowJo.

### Ultrasound Imaging

Ultrasound imaging was performed by Vevo 2100 high‐frequency ultrasound (FUJIFILM, VisualSonics Inc., Canada) with a 25‐MHz probe. Examination was performed using a contrast gain of 35 dB, dynamic range of 35 dB, and depth of 20 mm for in vivo, 15 mm for in vitro. Vevo anesthesia system was used for the maintenance of anesthesia. After the examination, ImageJ was used for data analysis.

### Statistical Analysis

One‐way ANOVA, two‐way ANOVA, and *t*‐tests were performed using GraphPad Prism 8 software (GraphPad Software Inc., USA) as statistical analysis. *P* value of < 0.05 was regarded as statistically significant.

### Ethics Approval Statement

All animal studies were approved by the Institutional Animal Care and Use Committee.

## Conflict of Interest

The authors declare no conflict of interest.

## Supporting information

Supporting Information

Supplemental Video 1

Supplemental Video 2

## Data Availability

The data that support the findings of this study are available from the corresponding author upon reasonable request.
