# Supplementary material for: Truncated PD1 Engineered Gas‐Producing Extracellular Vesicles for Ultrasound Imaging and Subsequent Degradation of PDL1 in Tumor Cells
Source: Adv Sci (Weinh). 2024 Jan 23;11(12):2305891. doi: 10.1002/advs.202305891 (PMC10966526; doi:10.1002/advs.202305891)
Supplement: Supplementary file 1 — Supporting Information [file ADVS-11-2305891-s001.pdf]

ADVANCED SCIENCE
Open Access

# Supporting Information

for *Adv. Sci.*, DOI 10.1002/advs.202305891

Truncated PD1 Engineered Gas-Producing Extracellular Vesicles for Ultrasound Imaging and Subsequent Degradation of PDL1 in Tumor Cells

*Siyan Zhang, Yuan Liang, Panpan Ji, Rui Zheng, Fan Lu, Guangdong Hou, Guodong Yang* and Lijun Yuan**

# Supporting Information

## Truncated PD1 engineered gas-producing extracellular vesicles for ultrasound imaging and subsequent degradation of PDL1 in tumor cells

*Siyan Zhang, Yuan Liang, Panpan Ji, Rui Zheng, Fan Lu, Guangdong Hou, Guodong Yang*, and Lijun Yuan**

**Table. S1 Plasmid sequences used in this study**

| **Sequences** | |
|---|---|
| tPd1-Ptgfrn-Flag | GCCACCATGGGGCGCCTGGCCTCGAGGCCGCTGCTGCTGGCGCTC<br>CTGTCGTTGGCTCTTTGCCGAGGGCGTGTGCTAGAGGTCCCCAATG<br>GGCCCTGGAGGTCCCTCACCTTCTACCCAGCCTGGCTCACAGTGTC<br>AGAGGGAGCAAATGCCACCTTCACCTGCAGCTTGTCCAACTGGTC<br>GGAGGATCTTATGCTGAACTGGAACCGCCTGAGTCCCAGCAACCA<br>GACTGAAAAACAGGCCGCCTTCTGTAATGGTTTGAGCCAACCCGT<br>CCAGGATGCCCGCTTCCAGATCATACAGCTGCCCAACAGGCATGA<br>CTTCCACATGAACATCCTTGACACACGGCGCAATGACAGTGGCAT<br>CTACCTCTGTGGGGCCATCTCCCTGCACCCCAAGGCAAAAATCGA<br>GGAGAGCCCTGGAGCAGAGCTCGTGGTAACAGAGAGAATCCTGG<br>AGACCTCAACAAGATATCCCAGCCCCTCGCCCAAACCAGAAGGCC<br>GGTTTCAAGGCATGGGTGGCGGAGGCTCGGGCGGAGGTGGGTCGG<br>GTGGCGGCGGATCAGGTCCTATATTTAATGCTTCTGTGCATTCAGA<br>CACACCATCAGTAATTCGGGGAGATCTGATCAAATTGTTCTGTATC<br>ATCACTGTCGAGGGAGCAGCACTGGATCCAGATGACATGGCCTTT<br>GATGTGTCCTGGTTTGCGGTGCACTCTTTTGGCCTGGACAAGGCTC<br>CTGTGCTCCTGTCTTCCCTGGATCGGAAGGGCATCGTGACCACCTC<br>CCGGAGGGACTGGAAGAGCGACCTCAGCCTGGAGCGCGTGAGTGT<br>GCTGGAATTCTTGCTGCAAGTGCATGGCTCCGAGGACCAGGACTT<br>TGGCAACTACTACTGTTCCGTGACTCCATGGGTGAAGTCACCAAC<br>AGGTTCCTGGCAGAAGGAGGCAGAGATCCACTCCAAGCCCGTTTT<br>TATAACTGTGAAGATGGATGTGCTGAACGCCTTCAAGTATCCCTTG<br>CTGATCGGCGTCGGTCTGTCCACGGTCATCGGGCTCCTGTCCTGTC<br>TCATCGGGTACTGCAGCTCCCACTGGTGTTGTAAGAAGGAGGTTC<br>AGGAGACACGGCGCGAGCGCCGCAGGCTCATGTCGATGGAGATG |

| | |
|---|---|
| | GACGATTACAAGGATGACGACGATAAGGGAGATTACAAGGATGA<br>CGACGATAAGATCGATTACAAGGATGACGACGATAAGTAG |
| Pd1 | ATGTGGGTCCGGCAGGTACCCTGGTCATTCACTTGGGCTGTGCTGC<br>AGTTGAGCTGGCAATCAGGGTGGCTTCTAGAGGTCCCCAATGGGC<br>CCTGGAGGTCCCTCACCTTCTACCCAGCCTGGCTCACAGTGTCAGA<br>GGGAGCAAATGCCACCTTCACCTGCAGCTTGTCCAACTGGTCGGA<br>GGATCTTATGCTGAACTGGAACCGCCTGAGTCCCAGCAACCAGAC<br>TGAAAAACAGGCCGCCTTCTGTAATGGTTTGAGCCAACCCGTCCA<br>GGATGCCCGCTTCCAGATCATACAGCTGCCCAACAGGCATGACTT<br>CCACATGAACATCCTTGACACACGGCGCAATGACAGTGGCATCTA<br>CCTCTGTGGGGCCATCTCCCTGCACCCCAAGGCAAAAATCGAGGA<br>GAGCCCTGGAGCAGAGCTCGTGGTAACAGAGAGAATCCTGGAGA<br>CCTCAACAAGATATCCCAGCCCCTCGCCCAAACCAGAAGGCCGGT<br>TTCAAGGCATGGTCATTGGTATCATGAGTGCCCTAGTGGGTATCCC<br>TGTATTGCTGCTGCTGGCCTGGGCCCTAGCTGTCTTCTGCTCAACA<br>AGTATGTCAGAGGCCAGAGGAGCTGGAAGCAAGGACGACACTCT<br>GAAGGAGGAGCCTTCAGCAGCACCTGTCCCTAGTGTGGCCTATGA<br>GGAGCTGGACTTCCAGGGACGAGAGAAGACACCAGAGCTCCCTAC<br>CGCCTGTGTGCACACAGAATATGCCACCATTGTCTTCACTGAAGG<br>GCTGGGTGCCTCGGCCATGGGACGTAGGGGCTCAGCTGATGGCCT<br>GCAGGGTCCTCGGCCTCCAAGACATGAGGATGGACATTGTTCTTG<br>GCCTCTTTGA |
| Pdl1 | ATGAGGATATTTGCTGGCATTATATTCACAGCCTGCTGTCACTTGC<br>TACGGGCGTTTACTATCACGGCTCCAAAGGACTTGTACGTGGTGG<br>AGTATGGCAGCAACGTCACGATGGAGTGCAGATTCCCTGTAGAAC<br>GGGAGCTGGACCTGCTTGCGTTAGTGGTGTACTGGGAAAAGGAAG<br>ATGAGCAAGTGATTCAGTTTGTGGCAGGAGAGGAGGACCTTAAGC<br>CTCAGCACAGCAACTTCAGGGGGAGAGCCTCGCTGCCAAAGGACC<br>AGCTTTTGAAGGGAAATGCTGCCCTTCAGATCACAGACGTCAAGC<br>TGCAGGACGCAGGCGTTTACTGCTGCATAATCAGCTACGGTGGTG<br>CGGACTACAAGCGAATCACGCTGAAAGTCAATGCCCCATACCGCA<br>AAATCAACCAGAGAATTTCCGTGGATCCAGCCACTTCTGAGCATG<br>AACTAATATGTCAGGCCGAGGGTTATCCAGAAGCTGAGGTAATCT<br>GGACAAACAGTGACCACCAACCCGTGAGTGGGAAGAGAAGTGTC<br>ACCACTTCCCGGACAGAGGGGATGCTTCTCAATGTGACCAGCAGT<br>CTGAGGGTCAACGCCACAGCGAATGATGTTTTCTACTGTACGTTTT |

| | |
|---|---|
| | GGAGATCACAGCCAGGGCAAAACCACACAGCGGAGCTGATCATC<br>CCAGAACTGCCTGCAACACATCCTCCACAGAACAGGACTCACTGG<br>GTGCTTCTGGGATCCATCCTGTTGTTCCTCATTGTAGTGTCCACGG<br>TCCTCCTCTTCTTGAGAAAACAAGTGAGAATGCTAGATGTGGAGA<br>AATGTGGCGTTGAAGATACAAGCTCAAAAAACCGAAATGATACAC<br>AATTCGAGGAGACGTAA |
| Pdl1-Gfp | ATGAGGATATTTGCTGGCATTATATTCACAGCCTGCTGTCACTTGC<br>TACGGGCGTTTACTATCACGGCTCCAAAGGACTTGTACGTGGTGG<br>AGTATGGCAGCAACGTCACGATGGAGTGCAGATTCCCTGTAGAAC<br>GGGAGCTGGACCTGCTTGCGTTAGTGGTGTACTGGGAAAAGGAAG<br>ATGAGCAAGTGATTCAGTTTGTGGCAGGAGAGGAGGACCTTAAGC<br>CTCAGCACAGCAACTTCAGGGGGAGAGCCTCGCTGCCAAAGGACC<br>AGCTTTTGAAGGGAAATGCTGCCCTTCAGATCACAGACGTCAAGC<br>TGCAGGACGCAGGCGTTTACTGCTGCATAATCAGCTACGGTGGTG<br>CGGACTACAAGCGAATCACGCTGAAAGTCAATGCCCCATACCGCA<br>AAATCAACCAGAGAATTTCCGTGGATCCAGCCACTTCTGAGCATG<br>AACTAATATGTCAGGCCGAGGGTTATCCAGAAGCTGAGGTAATCT<br>GGACAAACAGTGACCACCAACCCGTGAGTGGGAAGAGAAGTGTC<br>ACCACTTCCCGGACAGAGGGGATGCTTCTCAATGTGACCAGCAGT<br>CTGAGGGTCAACGCCACAGCGAATGATGTTTTCTACTGTACGTTTT<br>GGAGATCACAGCCAGGGCAAAACCACACAGCGGAGCTGATCATC<br>CCAGAACTGCCTGCAACACATCCTCCACAGAACAGGACTCACTGG<br>GTGCTTCTGGGATCCATCCTGTTGTTCCTCATTGTAGTGTCCACGG<br>TCCTCCTCTTCTTGAGAAAACAAGTGAGAATGCTAGATGTGGAGA<br>AATGTGGCGTTGAAGATACAAGCTCAAAAAACCGAAATGATACAC<br>AATTCGAGGAGACGATGGTGAGCAAGGGCGAGGAGCTGTTCACC<br>GGGGTGGTGCCCATCCTGGTCGAGCTGGACGGCGACGTAAACGGC<br>CACAAGTTCAGCGTGTCCGGCGAGGGCGAGGGCGATGCCACCTAC<br>GGCAAGCTGACCCTGAAGTTCATCTGCACCACCGGCAAGCTGCCC<br>GTGCCCTGGCCCACCCTCGTGACCACCCTGACCTACGGCGTGCAG<br>TGCTTCAGCCGCTACCCCGACCACATGAAGCAGCACGACTTCTTCA<br>AGTCCGCCATGCCCGAAGGCTACGTCCAGGAGCGCACCATCTTCT<br>TCAAGGACGACGGCAACTACAAGACCCGCGCCGAGGTGAAGTTCG<br>AGGGCGACACCCTGGTGAACCGCATCGAGCTGAAGGGCATCGAC<br>TTCAAGGAGGACGGCAACATCCTGGGGCACAAGCTGGAGTACAAC<br>TACAACAGCCACAACGTCTATATCATGGCCGACAAGCAGAAGAAC |

| | |
|---|---|
| | GGCATCAAGGTGAACTTCAAGATCCGCCACAACATCGAGGACGGC AGCGTGCAGCTCGCCGACCACTACCAGCAGAACACCCCCATCGGC GACGGCCCCGTGCTGCTGCCCGACAACCACTACCTGAGCACCCAG TCCGCCCTGAGCAAAGACCCCAACGAGAAGCGCGATCACATGGTC CTGCTGGAGTTCGTGACCGCCGCCGGGATCACTCTCGGCATGGAC GAGCTGTACAAGTAA |

**Table. S2 Primers used in this study**

| **Primer** | **Forward** | **Reverse** |
|---|---|---|
| *tPd1-Ptgfrn* | 5’CTGTCGTTGGCTCTTTGCCG3’ | 5’CAGGCTGGGTAGAAGGTGAG3’ |
| *Pdl1* | 5’GCCTGCTGTCACTTGCTACG3’ | 5’GTCCAGCTCCCGTTCTACAG3’ |
| *Pd1* | 5’GAAAAACAGGCCGCCTTCTG3’ | 5’CCTGTTGGGCAGCTGTATGA3’ |
| *Il6* | 5’AGTCCTTCCTACCCCAATTTCC3’ | 5’TGGTCTTGGTCCTTAGCCAC3’ |
| *Ifnγ* | 5’AGCAAGGCGAAAAAGGATGC3’ | 5’TCATTGAATGCTTGGCGCTG3’ |
| *Tnfα* | 5’GATCGGTCCCCAAAGGGATG3’ | 5’CCACTTGGTGGTTTGTGAGTG3’ |
| *Gapdh* | 5’AGGTCGGTGTGAACGGATTTG3’ | 5’GGGGTCGTTGATGGCAACA3’ |
| hGAPDH | 5’CCACCACACTGAATCTCCCC3’ | 5’TGGTTGAGCACAGGGTACTT3’ |

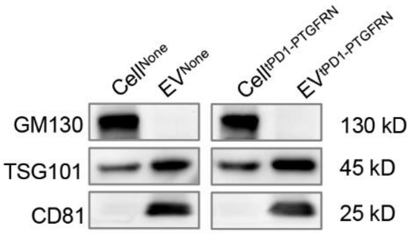


**Figure S1. Western blot analysis of the exosomal inclusive and exclusive markers in EVs.** GAPDH served as a loading control.

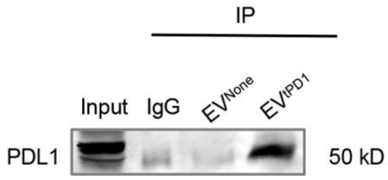


**Figure S2. Immunoprecipitation analysis of the interaction between PDL1 and tPD1 on EV.** Diluted 4T1-Pdl1 cell lysates were incubated with indicated EVs, followed by EV isolation. Immunoprecipitation with an unrelated IgG serves as negative control.

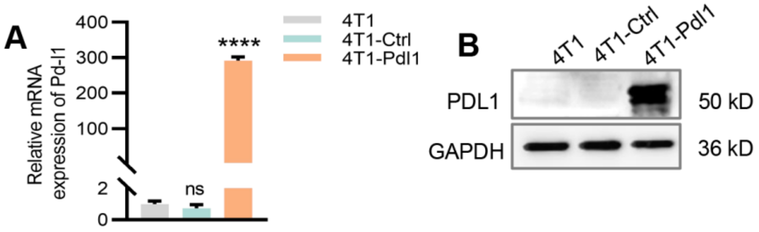


**Figure S3. Construction of Pdl1 overexpressing 4T1 cells.** A) qPCR analysis of Pdl1 mRNA expression. Cells were untreated or infected with control or Pdl1 overexpressing virus. *ns*: not significant; ****$P < 0.0001$ by *One-way ANOVA*. B) Western blot analysis of PDL1 expression. GAPDH served as a loading control.

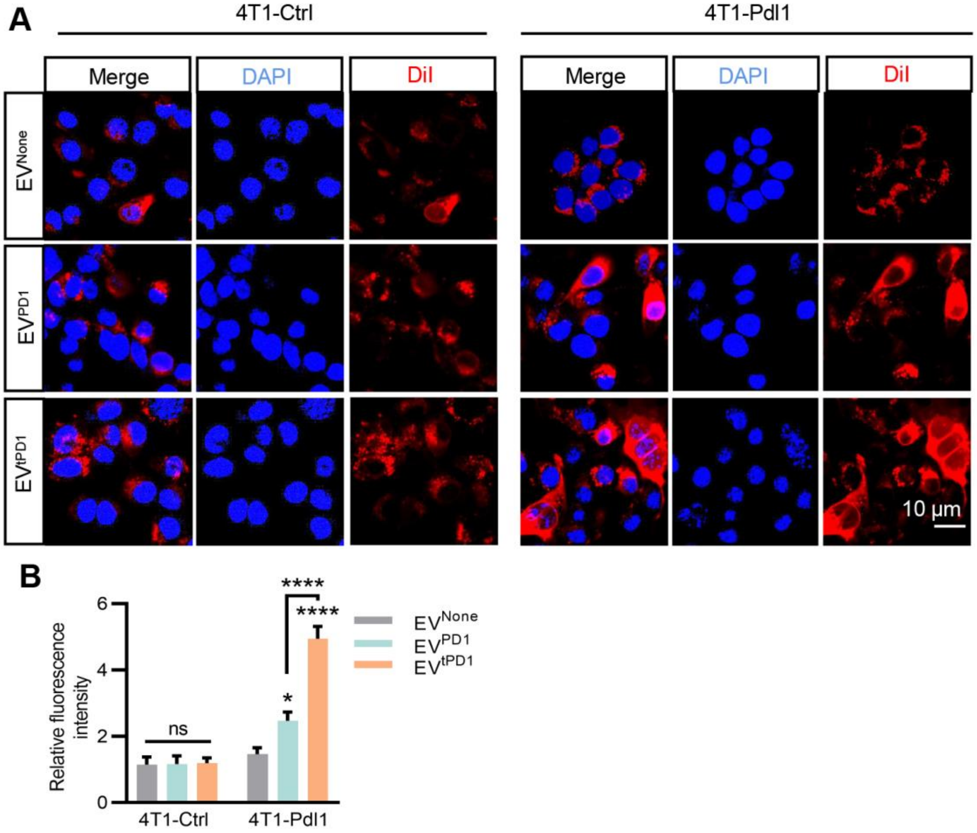


**Figure S4. Uptake of EV$^{tPD1}$ by cells infected with 4T1-Ctrl or 4T1-Pdl1 virus.** A) Pdl1 was overexpressed in 4T1 cells. 4T1 cells infected with control (4T1-Ctrl) or Pdl1 (4T1-Pdl1) overexpressing lentivirus were co-cultured with DiI labeled EVs for 4 h. Nuclei were stained with DAPI (blue). Scale bar = 10 µm. B) Quantification of immunofluorescence signal. *ns*: not significant; $*P < 0.05$; $****P < 0.0001$ by *One-way ANOVA*. Data shown are representative of 3 different experiments.

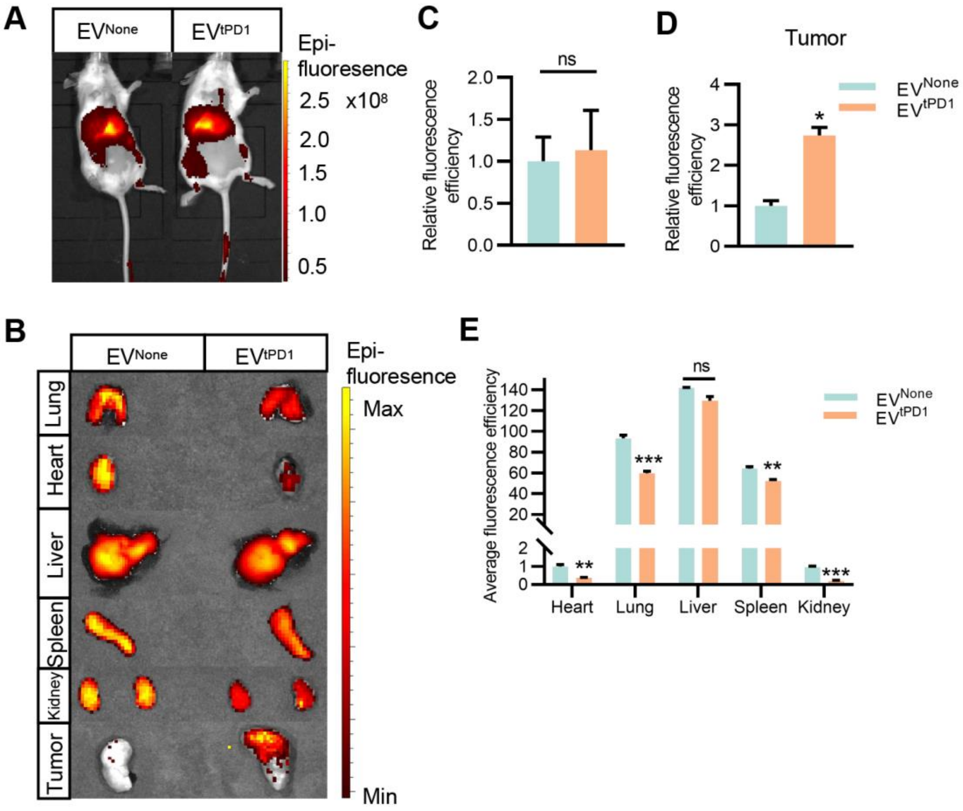


**Figure S5. Distribution of EV$^{tPD1}$ in vivo.** A, B) In vivo and ex vivo fluorescence imaging analyses. C) Quantification of the in vivo fluorescence intensity. D) Quantification of the fluorescence intensity in tumors. E) Quantification of the fluorescence intensity in different organs. *ns*: not significant; *$P<0.05$; **$P<0.01$; ***$P<0.001$ by *t-test*.

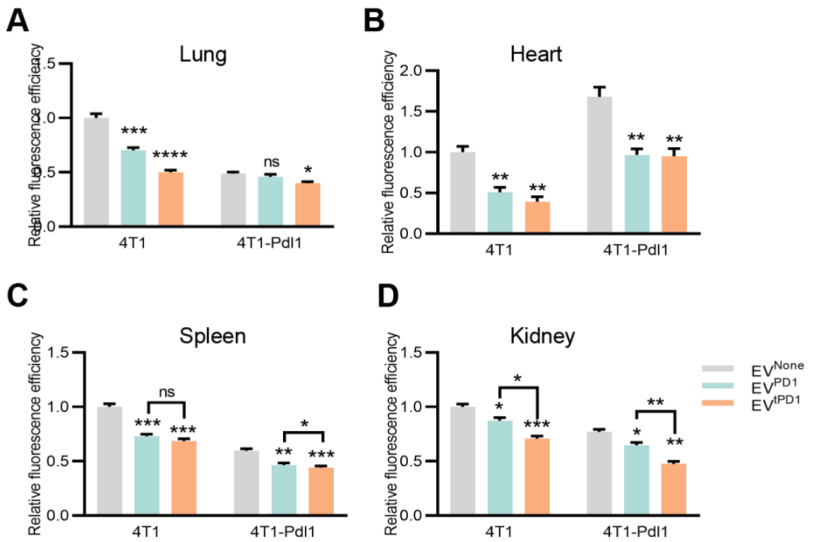


**Figure S6. Ex vivo distribution of EV$^{tPD1}$in main organs of 4T1-Pdl1 tumor model.** A-D) Quantification of the fluorescence intensity different organs. Data were expressed as the mean of 3 mice in each group. *ns*: not significant; **P*<0.05; ***P*<0.01; ****P*<0.001; *****P*<0.0001 by *One-way ANOVA*.

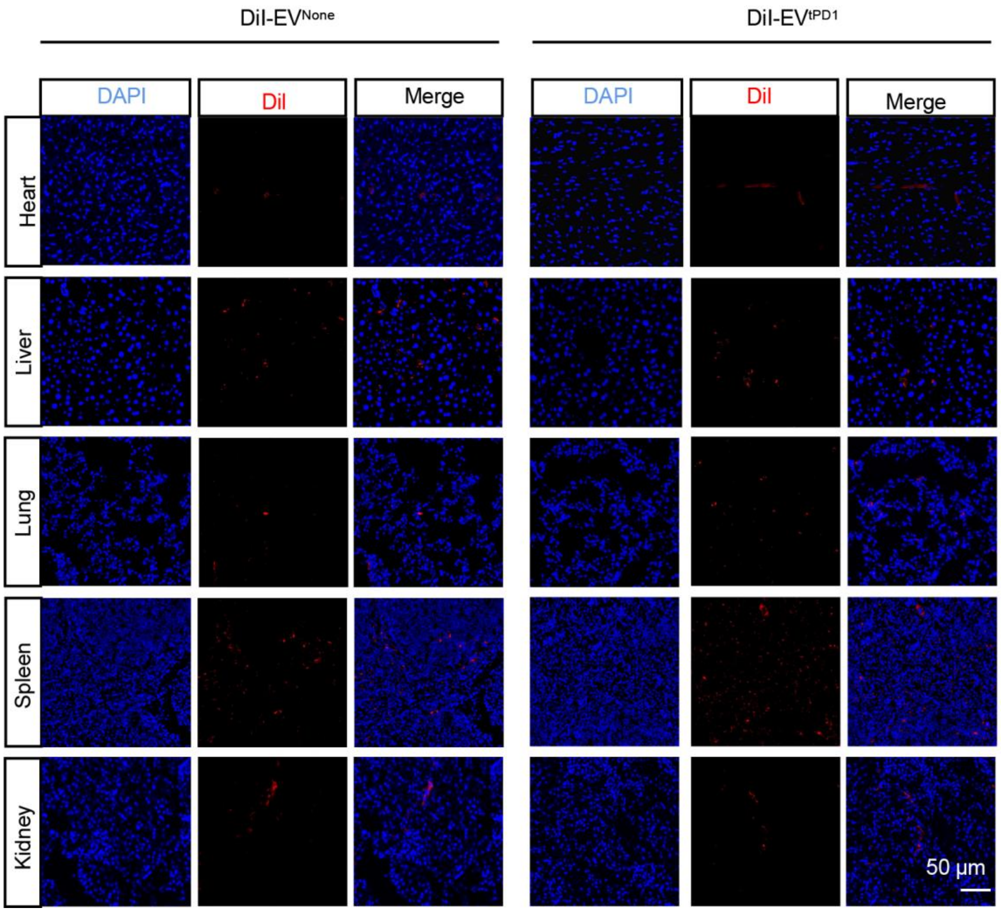


**Figure S7. Fluorescence microscopy analysis of distribution of engineered EVs in different organs.** Fluorescence microscopy images of the DiI (red) labeled EVs in different organs. Nuclei were counterstained with DAPI (blue). Representative images of the data from 5 mice. Scale bar = 50 μm.

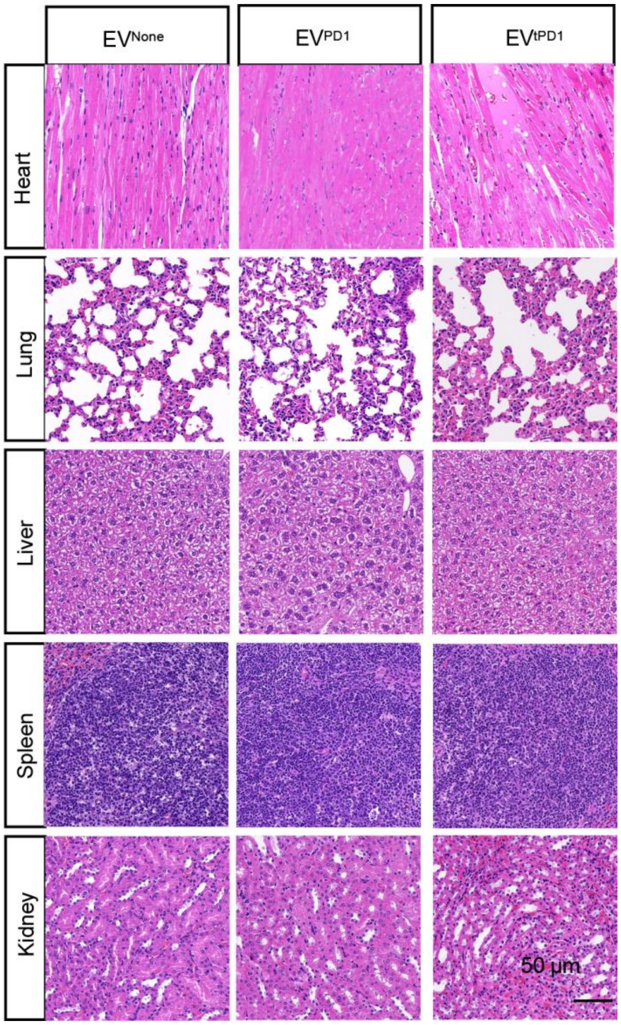


**Figure S8. Histological analysis of the side-effects of EV treatments.** H&E staining of different organs in mice treated as indicated. Scale bar = 100 μm. Data shown are representative of 5 mice in each group.

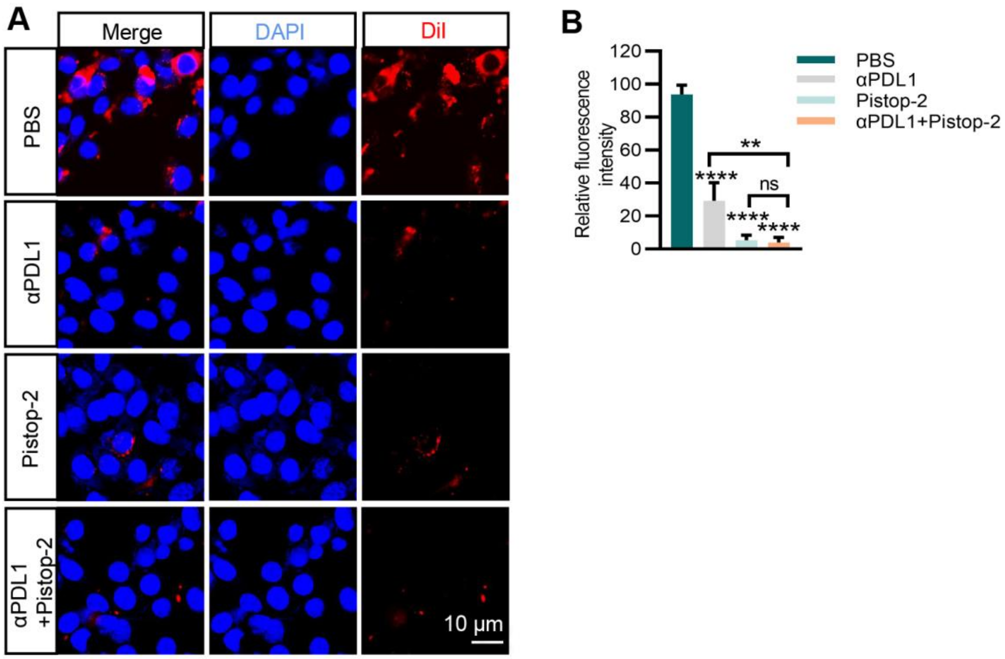


**Figure S9. Uptake of EVs by 4T1-Pdl1 cells in a receptor dependent endocytosis.** A) 4T1-Pdl1 cells were treated with EV$^{tPD1}$ alone or together with anti-PDL1 antibody (7.5 μg/ml), Psitop-2 (25 μM) or combination. EVs were labeled with DiI (red), and nuclei were stained with DAPI (blue). Data shown are representative of 3 different experiments. Scale bar = 10 μm. B) Quantification data. *ns*: not significant; *****P <0.0001* by *One-way ANOVA.*

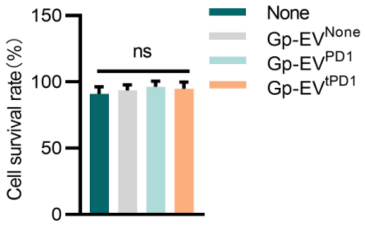


**Figure S10. Cell survival rate of 4T1-Pdl1 cells subjected to Gp-EVs treatment for 24 h.** Data shown are representative of 3 different experiments. Statistical quantification of the intensity by *t-test*. *ns*: not significant.

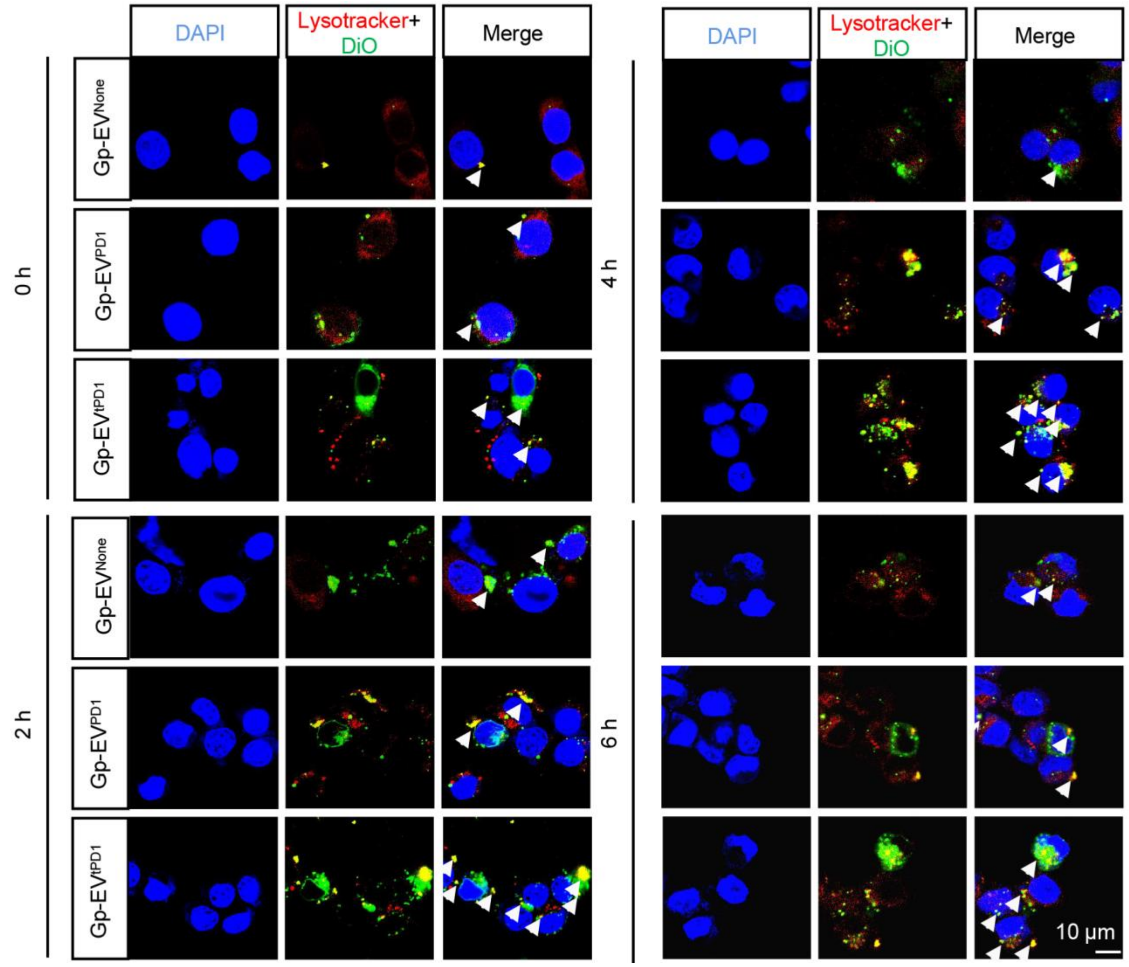


**Figure S11. Time-dependent co-localization of EVs and lysosomes.** Fluorescence co-localization stain images of the EVs and lysosomes. EVs were labeled with DiO (green). Lysosomes were stained with LysoTracker Red (red). Nuclei were stained with DAPI (blue). Data shown are representative of 3 different experiments. Scale bar = 10 μm.

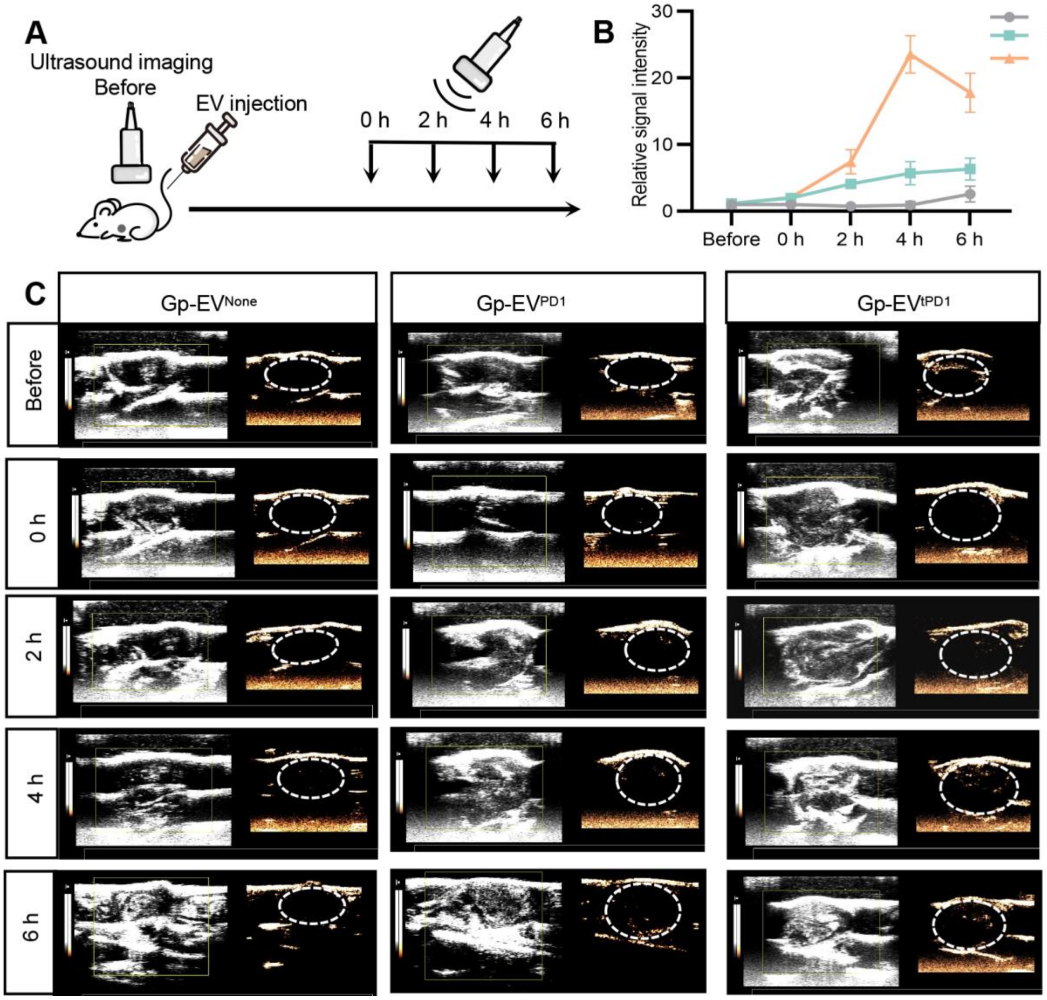


**Figure S12. Time-dependent ultrasound imaging changes in 4T1 syngeneic model receiving different Gp-EV treatments.** A) Schematic illustration of the experimental procedure. Female Balb/c mice aged 6-8 weeks were orthotopically implanted with 4T1-Pdl1 cells. After 14 days, mice were injected with control, Gp-EV$^{PD1}$ or Gp-EV$^{tPD1}$ via tail veins. US images were harvested before and after injection for 0, 2, 4, and 6 hours. B-C) In vivo tumor US imaging of control, Gp-EV$^{PD1}$ or Gp-EV$^{tPD1}$. ****$P < 0.0001$ by *Two-way ANOVA*.

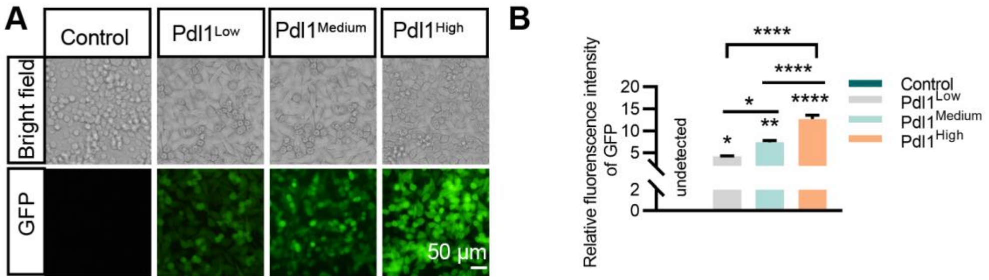


**Figure S13. GFP intensity in 4T1 cells with gradient PDL1 expression levels.** A-B) Fluorescence microscopy analysis of GFP expression in cells after infection. Scale bar = 50 μm. Statistical quantification of the intensity by *One-way ANOVA*. *$P<0.05$; **$P<0.01$; ****$P<0.0001$;

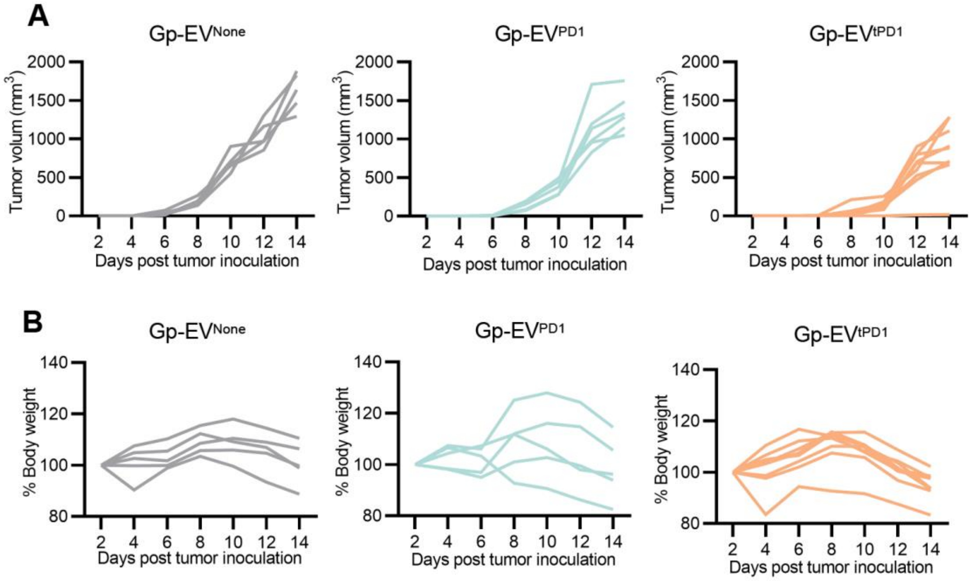


**Figure S14. Individual growth curves of the 4T1-Pdl1$^{High}$ syngeneic model receiving indicated treatments.** A) Individual tumor volume curves of the 4T1-Pdl1$^{High}$ model. B) Individual body weight curves for the 4T1-Pdl1$^{High}$ tumor-bearing mice treated as indicated. Data shown are representative of 5-7 mice in each group.

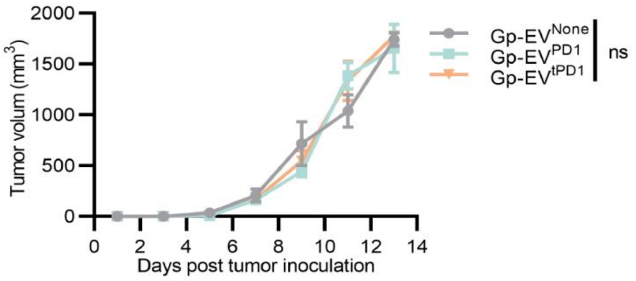


**Figure S15. Tumor growth in 4T1-Pdl1$^{High}$ inoculated nude mice.** Balb/c nude mice were implanted 4T1-Pdl1$^{High}$ cells, followed by treatment with control, Gp-EV$^{PD1}$ or Gp-EV$^{tPD1}$. Data shown are representative of 5-7 mice in each group. *ns*: not significant by *Two-way ANOVA*.

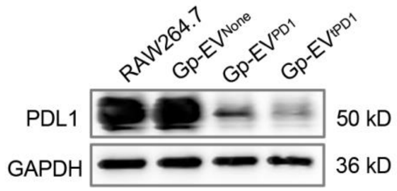


**Figure S16. Western blot analysis of PDL1 expression in RAW267.4 cells treated with indicated Gp-EVs.** GAPDH served as a loading control.

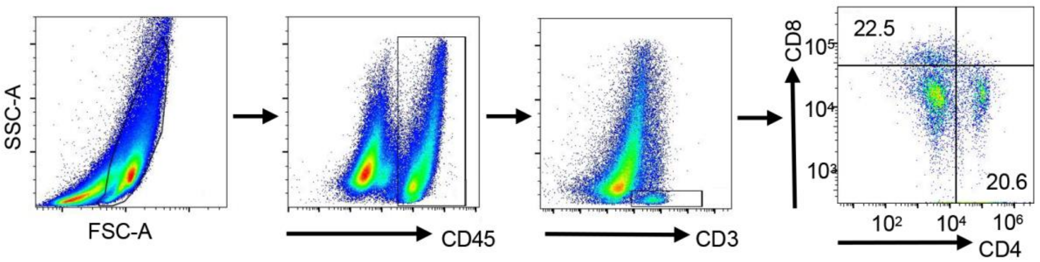


**Figure S17. Gating strategy for CD4+ and CD8+ T cells.**

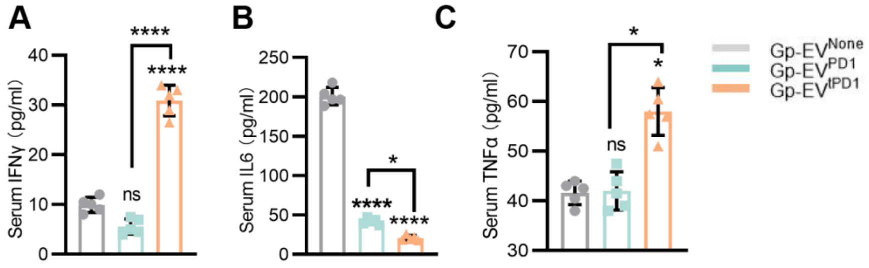


**Figure S18. The levels of the pro-inflammatory cytokines in the serum.** The levels of IFNγ (A), IL6 (B), and TNFα (C) in the serum of 4T1-Pdl1$^{High}$ tumor-bearing mice. Data shown are representative of 5 mice in each group. *ns*: not significant; *$P<0.05$; ****$P<0.0001$ by *One-way ANOVA.*
